# A comparison of Direct sequencing, Pyrosequencing, High resolution melting analysis, TheraScreen DxS, and the K-ras StripAssay for detecting *KRAS* mutations in non small cell lung carcinomas

**DOI:** 10.1186/1756-9966-31-79

**Published:** 2012-09-20

**Authors:** Sylwia Jancik, Jiri Drabek, Jitka Berkovcova, Yong Zhong Xu, Marcela Stankova, Jiri Klein, Vitezslav Kolek, Josef Skarda, Tomas Tichy, Ivona Grygarkova, Danuta Radzioch, Marian Hajduch

**Affiliations:** 1 Laboratory of Experimental Medicine, Institute of Molecular and Translational Medicine, Faculty of Medicine and Dentistry, Palacky University and University Hospital in Olomouc, Olomouc, Czech Republic; 2 IntellMed Ltd, The Science and Technology Park of Palacky University, Olomouc, Czech Republic; 3 Departments of Experimental Medicine and Human Genetics, McGill University, Montreal, Quebec, Canada; 4 Institute of Applied Biotechnologies, Prague, Czech Republic; 5 1st Department of Surgery, Faculty of Medicine and Dentistry, Palacky University and University Hospital in Olomouc, Olomouc, Czech Republic; 6 Department of Pneumology and Tuberculosis, Faculty of Medicine and Dentistry, Palacky University and University Hospital in Olomouc, Olomouc, Czech Republic; 7 Laboratory of Molecular Pathology, Institute of Molecular and Translational Medicine, Faculty of Medicine and Dentistry, Palacky University and University Hospital in Olomouc, Olomouc, Czech Republic

**Keywords:** SNP - single nucleotide polymorphism, KRAS - Kiras2 kristen rat sarcoma viral oncogene homolog, NSCLC - Non-small cell lung cancer, Genotyping

## Abstract

**Background:**

It is mandatory to confirm the absence of mutations in the *KRAS* gene before treating metastatic colorectal cancers with epidermal growth factor receptor inhibitors, and similar regulations are being considered for non-small cell lung carcinomas (NSCLC) and other tumor types. Routine diagnosis of *KRAS* mutations in NSCLC is challenging because of compromised quantity and quality of biological material. Although there are several methods available for detecting mutations in *KRAS*, there is little comparative data regarding their analytical performance, economic merits, and workflow parameters.

**Methods:**

We compared the specificity, sensitivity, cost, and working time of five methods using 131 frozen NSCLC tissue samples. We extracted genomic DNA from the samples and compared the performance of Sanger cycle sequencing, Pyrosequencing, High-resolution melting analysis (HRM), and the Conformité Européenne (CE)-marked TheraScreen DxS and K-ras StripAssay kits.

**Results and conclusions:**

Our results demonstrate that TheraScreen DxS and the StripAssay, in that order, were most effective at diagnosing mutations in *KRAS*. However, there were still unsatisfactory disagreements between them for 6.1% of all samples tested. Despite this, our findings are likely to assist molecular biologists in making rational decisions when selecting a reliable, efficient, and cost-effective method for detecting *KRAS* mutations in heterogeneous clinical tumor samples.

## Background

At present, identifying targeted anticancer treatment suitable for a given patient requires the availability of accurate diagnostics. Diagnostic techniques therefore have a significant impact on patients’ survival and quality of life [1]. In recent years, it has become apparent that certain types of tumors undergo mutations that either originate from the aberrant physiology of the tumor or are induced/selected by mutagenic cancer therapies [2-4]. Failure to detect mutations in important regulatory genes in tumor specimens may have serious consequences for the patients, because these alterations can significantly reduce the effectiveness of certain biological and cytotoxic therapies. Mutations in the *KRAS* oncogene are often found in human cancers. They are most common in pancreatic cancer, which can exhibit mutation rates of 80 - 90%. *KRAS* mutations are also observed in 40 – 50% of colorectal cancers and 10 - 30% of Non-Small Cell Lung Cancers (NSCLCs).

Recent studies have shown that some anticancer drugs are only effective against tumors in which the KRAS signaling pathway has not undergone oncogenic activation. These include the small-molecule epidermal growth factor receptor inhibitors erlotinib (Tarceva®) and gefitinib (Iressa®), which are used to treat NSCLC patients, and monoclonal antibody therapies such as cetuximab (Erbitux®) and panitumumab (Vectibix®), which are primarily used in the treatment of metastatic colorectal cancers (mCRC) [5-7]. According to the U.S. National Comprehensive Cancer Network (NCCN) guidelines from November 2008 ( http://www.nccn.org/about/news/newsinfo.asp?NewsID=194) and recommendations of the American Society of Clinical Oncology (ASCO) [8], screening of the status of the *KRAS* gene is mandatory when deciding whether or not a patient with colorectal cancer should receive anti-EGFR drugs. Similar rules are being considered for NSCLC where *KRAS* mutations have prognostic value for progressive disease in adenocarcinoma [9,10].

There are multiple methods for detecting *KRAS* mutations in patient tissues, with varying analytical parameters. Individual methods need to be evaluated in terms of their sensitivity, specificity, and cost per analysis before they can be considered to meet acceptable gold standards in clinical practice. A standardized European quality assurance program for tests to detect mutations in *KRAS* was proposed at the Third International Congress of Pathology, held by the European Society of Pathology (ESP) in Barcelona in May 2008. This program is focused on achieving optimal accuracy and proficiency across the European Union [11]. However, there are many methods in current use, some of which are only employed by individual laboratories and are not commercially available. These typically include sequencing assays [12] and gel-based DNA conformation assays [13,14]. Some of the commercial assays for detecting mutations in the *KRAS* gene have not yet been validated for clinical use (i.e.: Allele-specific oligonucleotide hybridization - Invigene®, KRAS mutation test kit - EntroGen®). At the time of writing, only the TheraScreen® kit sold by QiaGen, the KRAS LightMix® kit sold by TIB MolBiol, and the K-ras StripAssay® sold by ViennaLab had received the Conformité Européenne (CE) mark certifying them as being suitable for diagnostic use in the clinic under the terms of the European IVD Directive 98/79/EC.

In order to assess the specificity, sensitivity, cost, and working time of five frequently used methods for detecting mutations in *KRAS*, we performed parallel tests using DNA extracted from 131 frozen NSCLC tissue samples. The methods examined were Sanger cycle sequencing, Pyrosequencing, High-resolution melting analysis (HRM), and the CE-marked TheraScreen DxS and K-ras StripAssay kits. Our data demonstrate that there are important differences between these methods, which should be considered in routine clinical testing for *KRAS* mutations.

## Methods

### Pathological assessment

The experimental research presented in this manuscript was performed in compliance with the Helsinki Declaration according to the study ethics proposal approved by Ethical Board of Palacky University in Olomouc. Written informed consent was obtained from all patients for the use of the collected samples in the research projects which includes studies for publication of this report or any accompanied images.

Diagnosis of NSCLC was initially performed at the time of surgery and later confirmed from leftover by histological subtyping performed by experienced pathologist. All samples were found to contain more than 70% of tumour cells from at least 200 cells.

### DNA extraction from cell lines and primary tumor samples

Genomic DNA was extracted from 131 frozen Non Small Cell Lung Cancer (NSCLC) tissue specimens removed from patients undergoing surgery for lung cancer. Tissue was snap frozen in liquid nitrogen immediately after surgery and stored at −80°C until analyzed. Cell lines with specific *KRAS* mutations were obtained from the American Tissue Culture Collection (ATCC, Rockville, MA) and cultured according to ATCC instructions. DNA extraction and purification was performed using the QIAquick (QIAGEN, Hilden, Germany) isolation kit according to manufacturer’s instructions; in each case examined, the five methods were tested against the same DNA isolate, so potential differences in percentage of tumor cells does not confound the method comparison. Concentrations of DNA samples were measured spectrophotometrically using a NanoDrop ND 1000 spectrophotometer (NanoDropTechnologies, Wilmington, USA).

### Genotyping methods

Analyses were performed according to a blinded design, in which the experimentalist was not aware of the *KRAS* mutation status of any given sample. 131 NSCLC samples were analyzed using four methods: Direct sequencing, Pyrosequencing, and the TheraScreen DxS and K-ras StripAssay kits. Due to limited amount of tissue, only 116 samples from this group were also subjected to HRM analysis and 114 yielded usable data. Significance of the concordance of mutation detection with different methods for two categories (wildtype and mutant) was assessed by κ statistics ( http://faculty.vassar.edu/lowry/kappa.html).

### Direct sequencing method

**Figure 1:**
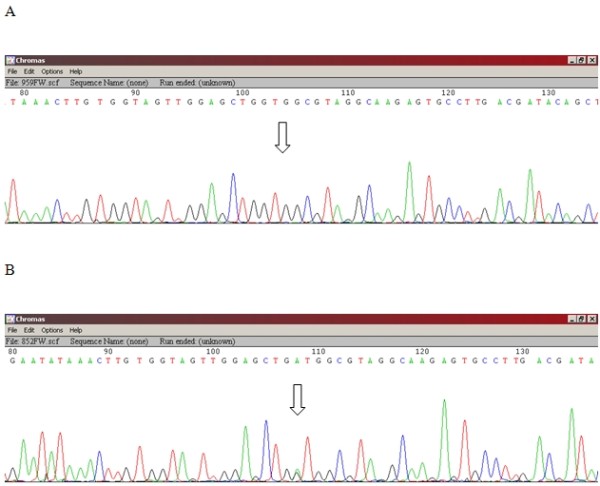
**Sequencing of the KRAS gene in DNA isolated from NSCLC tissues.** (**A**) Wild type-(12Gly-GGT, 13Gly-GGC), (**B**) Mutant- (12Asp-GAT).

Two primers were used to prepare amplicons for use in Sanger dideoxy termination sequencing [15]: a forward (FW) primer, 5'AAA AGG TAC TGG TGG AGT ATT TGA, and a 3’ reverse (REV) primer, 5' TCA TGA AAA TGG TCA GAG AAA CC 3' (Generi-Biotech, Hradec Králové, Czech Republic). PCR was performed with a reaction volume of 50 μl in an MJ Research PTC-200 Peltier Thermal Cycler (Watertown, USA). The composition of the PCR reaction mixture was as follows: MgCl_2_ (3 mM, ThermoScientific, Waltham, USA), dNTPs (0.2 mM, ThermoScientific), ThermoStart DNA polymerase (2U, ThermoScientific), FW-primer (0.3 μM), REV-primer (0.3 μM), 1xPCR buffer, and between 10 ng and 100 ng of genomic DNA per reaction. The following amplification program was used: 95°C/15 min to activate the Taq polymerase; 35x (95°C/30 s, 58°C/30 s 72°C/30 s) for denaturation, annealing, and extension; and finally 75°C/5 min to finalize the extension, followed by cooling to 15°C. The PCR product was separated using a 2% agarose gel and purified using the QIAquick PCR purification kit (QIAGEN, Hilden, Germany). For each sample specimen, separate sequencing reactions were performed using the forward (FW) and reverse (REV) primers. The sequencing primers were internal to the amplicons from the previous PCR cycles: FW - 5' TTA ACC TTA TGT GTG ACA TGT TCT AA 3', REV - 5' AGA ATG GTC CTG CAC CAG TAAT 3'. Sequencing reactions were performed according to the manufacturer’s protocol in a 20 μl reaction volume containing 4 μl DTCS Quick Start kit (Beckman Coulter, Brea, USA), 1 μl (10 μM) of the FW or REV primer, 10 μl nuclease-free water, and 5 μl of 25x diluted template PCR product. After cleaning, precipitated DNA was diluted in SLS-formamide (Beckman Coulter, Brea, USA) and dideoxylabelled fragments were size-separated using an automated CEQ 8800 Genetic Analysis System (Beckman Coulter, Brea,USA) (Figure 1). Sequence identification was performed using version 1.42 of the Chromas software package (Conor McCarthy, Southport, Australia). For all analyses, data obtained with the forward and reverse primers were combined and aligned to the consensus sequence obtained from the BLAST GenBank database http://www.ncbi.nlm.nih.gov/nuccore/166706780?report=genbank.

### Pyrosequencing

In the pyrosequencing method for DNA sequence analysis [16,17], inorganic phosphate released in the course of nucleotide incorporation serves as the initial substrate in a sequence of four successive enzymatic reactions. This result in the emission of light, which functions as a signal that is proportional to the number of nucleotides incorporated.

**Figure 2:**
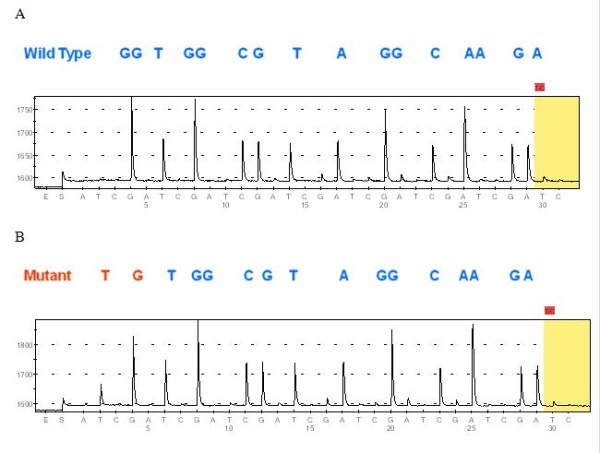
**Pyrosequencing of the KRAS gene in DNA isolated from NSCLC tissues.** (**A**) Wild type-(12Gly-GGT, 13Gly-GGC), (**B**) Mutant-*KRAS* (12Cys-TGT).

In this project, the PyroMark K-ras assay test (Biotage, Uppsala, Sweden) was used for primary amplification and pyrosequencing of both the 12th and the 13th codons of the *KRAS* oncogene (Figure 2). The following amplification program was used: the mixture was heated at 95°C for 5 min, then subjected to 45 cycles of 95°C for 15 s, 57°C for 30 s, and 72°C for 15 s. It was then held at 72°C for 5 min, and finally cooled to and held at 4°C. The final concentrations of the PCR components were: 1x PCR buffer, 2 mM MgCl_2_, 0.125 mM dNTPs, 0.2 μM FW primer and 0.2 μM REV biotinylated primer, 1U of AmpliTaq polymerase (Perkin Elmer, Waltham, USA) and 2 ng/μl DNA template. Fifteen μl of the PCR product was run on a 1,5% agarose gel (Sigma-Aldrich, St. Louis, USA) to confirm successful amplification, and 100 ng of PCR products were sent to the EpigenDX company (Worcester, USA) to be analyzed using the PyroMark MD System and the Pyromark ID analysis Software with previously validated cut-off of 5%.

### K-RAS TheraScreen DxS

**Figure 3:**
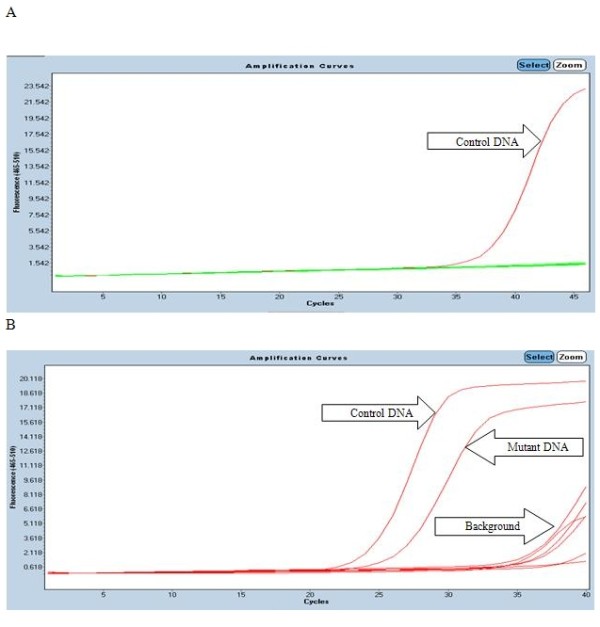
**TheraScreen analysis of the *KRAS *gene in DNA isolated from NSCLC tissue.** (**A**) Wild type. The figure shows a positive PCR control in which the quantity and quality of the DNA were assessed (red curve) without any other amplification of ARMS mutation positive primers (green curve) (**B**) Mutant. The figure shows a positive PCR control and a mutation signal (12Asp) generated by one tube of the ARMS-primers. The upper limit on ΔCt, which corresponds to a mutant DNA content of 1%, is for the mutant PCR to be 8 cycles behind the control PCR (here ΔCt = 26.44 - 24.03 = 2.41).

The TheraScreen DxS KRAS Mutation Kits KR-21 and KR-22 (QiaGen, Hilden, Germany) are designed to detect six mutations in codon 12 (Gly > Ala, Asp, Arg, Cys, Ser, and Val) and one in codon 13 (Gly > Asp) of the *KRAS* oncogene. The primers used in the assay have two characteristic features: sequence-specific 3’ ends (which comprise the PCR-Amplification Refractory Mutation System, PCR-ARMS®) to identify specific mutations, and Real-time PCR-Scorpion® primer tags, which fluoresce when incorporated into double-stranded DNA (Figure 3). The commercial test kit includes an internal reaction control and a synthetic control template. The degree of mutation of *KRAS* is calculated on the basis of the difference between the control reaction and the allele-specific reaction in terms of the number of cycles required for the fluorescence of the reaction mixture to exceed the background level (Δ-C_T_) [18].

PCR reactions were performed according to the protocol recommended by the manufacturer (TheraScreen K-RAS Mutation Kit version DU001PE) using a LightCycler®480 II (Roche Applied Science, Penzberg, Germany), with a final reaction volume of 25 μl. An initial denaturation step at 95°C for 4 min was followed by 45 cycles of 95°C for 30 sec and 60°C for 1 min. Analysis was performed using a predefined absolute quantification algorithm implemented in the LightCycler Analysis Software 1.5.0 SP3 program (Roche Applied Science, Penzberg, Germany) and by visual inspection conducted by two different researchers.

### K-ras StripAssay

**Figure 4:**
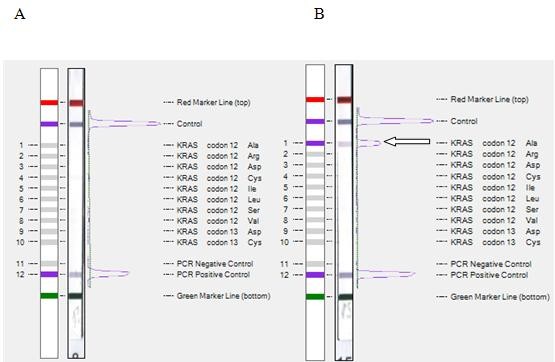
**StripAssay analysis of the *KRAS *gene in DNA isolated from NSCLC tissue.** (**A**) Wild type-(12Gly, 13Gly) (**B**) Mutant-(12Ala, 13Gly).

The K-ras StripAssay REF 5–590 (ViennaLab Diagnostics GmbH, Vienna, Austria) detects the 10 most common mutations in the *KRAS* gene by using multiplex mutant-enriched PCR and reverse-hybridization of the amplification products to nitrocellulose test strips (oligonucleotides used in the subsequent hybridization reactions are synthesized as probes targeting 8 mutations in codon 12 of the *KRAS* gene (Gly > Ala, Arg, Asp, Cys, Ile, Leu, Ser, and Val) and two mutations in codon 13 (Gly > Asp and Gly > Cys). Specifically hybridized biotinylated oligonucleotides are visualized using streptavidin-alkaline phosphatase and colored substrates (Figure 4).

The KRAS StripAssay was performed according to the manufacturer’s protocol (K-ras StripAssay™, ViennaLab Diagnostic GmbH, Vienna, Austria). Samples were diluted using deionized water to a concentration of 10 ng/μl. Five μl of diluted DNA was added to the multiplex PCR reaction with biotinylated primers, and PCR was conducted according to the manufacturer’s instructions. All of the incubation steps were performed using a PST-60 HL Plus thermoshaker (Biosan, Riga, Latvia) platform with the temperature set to 45°C. Scanning was performed using the EPSON Perfection V30 scanner (Epson America, Inc., Long Beach, USA) and bands were analyzed by StripAssayEvaluator software (ViennaLab, Vienna, Austria) and by visual inspection.

### High resolution melting analysis

**Figure 5:**
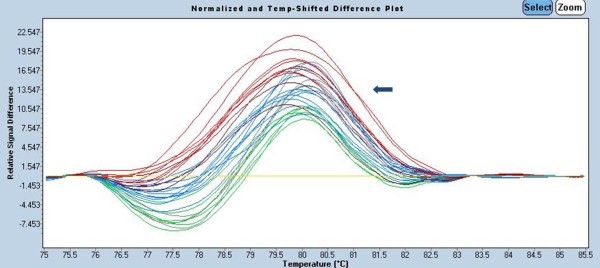
**HRM melting profile result of the *KRAS *gene isolated from NSCLC tissue.** The figure shows the results of Gene Scanning analyses (using the LC Green^PLUS^ dye with the sensitivity set to 0.3) of wild type samples (yellow line) and mutant cell line samples (curves with other than yellow colours).

The high-resolution melting (HRM) assay is a platform for real time detection of mutations that can be used to identify small differences in DNA sequences, even in heterozygous samples, by assessing changes in the shape of their melting curve profiles compared to profiles generated using standard (wild-type) DNA [19] (Figure 5). The HRM assay was developed using a new family of DNA-intercalating dyes including SYTO 9, LC Green and LC Green^PLUS+^ that display strong intercalation and even association with DNA.

HRM-PCR reaction components were used according to the instructions provided with the LightCycler® 480 II (Roche) instrument. The LC Green^PLUS^ (Idaho Technology, Salt Lake City, USA) intercalating dye was used, together with the primers FW-5'-AAA CTT GTG GTA GTT GGA GCT-3' (forward) and REV-5'-ATT AGC TGT ATC GTC AAG GCA-3' (reverse). The final concentrations of the components of the reaction mixture were: 1x PCR buffer, 2 mM MgCl_2,_ 0.2 μM dNTP, 0.5x LC Green^PLUS^, 0.2 U ThermoTaq polymerase (Thermo Scientific), and 0.3 μM FW and REV primer. The cycling and melting conditions were as follows: one cycle at 95°C for 15 min., followed by 45 cycles of 95°C for 10 s, 63°C for 10 s, and 72°C for 10 s. The sample was then melted by raising the temperature from 60°C to 95°C at a rate of 0.02 C/s. Mutations were analyzed using LightCycler Analysis Software 1.5.0 SP3 program (Roche Applied Science, Penzberg, Germany).

## Results

Five genotyping methods for determining the status of mutation of *KRAS* were assessed using frozen tissue from primary NSCLC tumor specimens. 131 DNA samples were analyzed with 4 of the methods (Direct sequencing, K-ras StripAssay, TheraScreen DxS, and Pyrosequencing), and 116 of these were also analyzed using the High resolution melting (HRM) technique. In the absence of a gold standard, we adopted a consensus method for assigning each sample’s mutation status. The results obtained and methodology used are shown in Table 1.

**Table 1:** Summary of the genotyping results obtained with the five tested methods in 131 NSCLC samples

**DNA sample number**	**Direct sequencing**	**Pyrosequencing**	**TheraScreen DxS**	**K-ras StripAssay**	**HRM**	**Consensus**
1	12Cys	12Cys	12Cys	12Cys	Mutation	12Cys
2	13Cys	13Cys	Wt	13Cys	Mutation	13Cys
3	12Cys	12Cys	12Val	Wt	Mutation	12Cys
4	12Asp	12Asp	12Asp	12Asp	Mutation	12Asp
5	12Cys	12Cys	12Cys	12Cys	Mutation	12Cys
6	12Cys	12Cys	12Cys	12Cys	Mutation	12Cys
7	Wt	12Cys	12Cys	12Cys	Mutation	12Cys
8	Wt	12Val	12Val	12Val	Mutation	12Val
9	Wt	12Cys	12Cys	12Cys	Mutation	12Cys
10	Wt	12Cys	12Cys	12Cys	Wt	12Cys
11	Wt	Wt	12Cys	12Cys	Wt	12Cys
12	Wt	Wt	12Cys	12Cys	Wt	12Cys
13	Wt	Wt	12Val	12Val	Wt	12Val
14	Wt	Wt	12Cys	12Cys	Wt	12Cys
15	Wt	Wt	12Cys	12Cys	Wt	12Cys
16	Wt	Wt	12Arg	13Cys	Inconclusive	Mutation
17	Wt	Wt	12Cys	12Cys	Mutation	12Cys
18	Wt	Wt	12Asp	13Asp	Not tested	Mutation
19	Wt	Wt	12Asp	Wt	Mutation	12Asp
20	Wt	Wt	12Cys	Wt	Not tested	Inconclusive
21	Wt	Wt	13Asp	Wt	Not tested	Inconclusive
22	Wt	Wt	Wt	12Cys	Mutation	12Cys
23	Wt	Wt	Wt	12Cys	Mutation	12Cys
24	Wt	Wt	Wt	12Arg	Wt	Wt
25	Wt	Wt	Wt	12Val	Wt	Wt
26	Wt	Wt	Wt	12Cys	Wt	Wt
27	Wt	Wt	Wt	13Cys	Wt	Wt
28	Wt	Wt	Wt	12Ala,13Cys	Wt	Wt
29	Wt	Wt	Wt	12Ser	Not tested	Inconclusive
30	Wt	Wt	Wt	12Ala	Wt	Wt
31	Wt	Wt	Wt	Wt	Mutation	Wt
32	Wt	Wt	Wt	Wt	Mutation	Wt
33 - 131	Wt (99 samples)	Wt (99 samples)	Wt (99 samples)	Wt (99 samples)	Wt (87 samples), 11-no DNA template, 1 –inconclusive sample.	Wt

The consensus result for a given sample was taken to be that obtained when the two CE-marked methods (K-ras StripAssay and TheraScreen DxS) were concordant with one-another (results that do not match this consensus are highlighted with a dark background). The detection of different types of mutation by different methods (e.g. in sample 3, p.Gly12Cys *vs* p.Gly12Val; in sample 16, p.Gly12Arg *vs* p.Gly13Cys; and in sample 18, p.Gly12Asp *vs* p.Gly13Asp) was not considered indicative of discrepancy because the precise identity of the mutation present is clinically irrelevant in this case (instances of type-of-mutation discordance are highlighted with a light background). In cases where the K-ras StripAssay and TheraScreen DxS kit generated inconsistent results, the sample was considered to be mutated only if one of the other three methods indicated the presence of a mutation. Thus, three samples (samples 20, 21, and 29) generated inconclusive results. Inconclusive results were excluded from further analysis.

As expected, the percentage of the DNA samples in which mutations were detected varied (from 20% to 5%) depending on the method of detection used. The Kras-StripAssay had the highest likelihood of referring a mutation in the *KRAS* locus, followed by TheraScreen DxS, HRM, Pyrosequencing, and Direct sequencing (Table 2).

**Table 2:** Number and percentage of mutations detected by methods

**Methods**	**Mutations/samples**	**%**	**Mutations/samples**	**%**
**Direct sequencing**	6/131	4.5	6/116	5.2
**Pyrosequencing**	10/131	7.6	10/116	8.7
**HRM**	-	-	15/116	13.1
**TheraScreen DxS**	20/131	15.2	17/116	14.6
**K-ras StripAssay**	26/131	19.8	24/116	20.7

To allow comparison with HRM, results are provided not only for 131 but also for 116 samples.

However, on the basis of our evaluation criteria (Table 1), the most sensitive tool was the TheraScreen DxS kit (95%), followed by the K-ras StripAssay (90%), HRM (70%), Pyrosequencing (48%), and Sequencing (29%). The most specific tools were the TheraScreen DxS kit, Sequencing, and Pyrosequencing (100%), followed by HRM (98%) and the K-ras StripAssay (95%) (Table 3).

**Table 3:** False positive and false negative rates of the different methods

	**Sequencing (n=131)**	**Pyrosequencing (n=131)**	**TheraScreen DxS (n=131)**	**K-ras StripAssay (n=131)**	**HRM (n=116)**
False positives (1 - specificity)	0/110 (0 %)	0/110 (0 %)	0/110 (0 %)	6/110 (5 %)	2/96 (2 %)
False negatives (1 - sensitivity)	15/21 (71 %)	11/21 (52 %)	1/21 (5 %)	2/21 (10 %)	6/20 (30 %)

**Figure 6:**
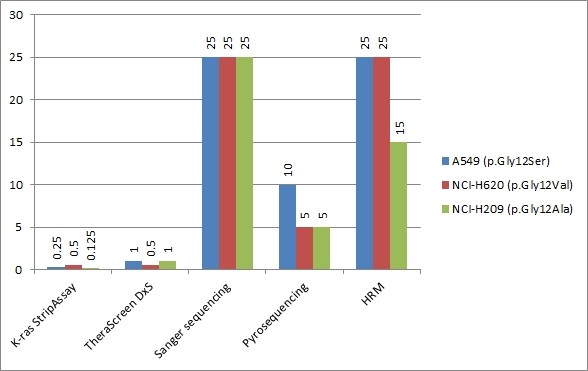
**Comparative sensitivity analysis of KRAS typing kits in dilution series, where DNA from three mutated cell lines was diluted in wild-type DNA.** Results of dilution series consisted of 25%, 20%, 15%, 10%, 5%, 1%, 0.5%, 0.25%, and 0.125% of mutated DNA in wild-type DNA. For threshold found in the first dilution experiment and one adjacent concentration from each side, typing was performed three times. Resulting consensus thresholds (found two or three times out of three repeats) for cell lines A549 (p.Gly12Ser), NCI-H620 (p.Gly12Val), and NCI-H209 (p.Gly12Ala) are shown in the graph.

The number of false positives and false negatives obtained with each method would change if one were to change the interpretation criteria. For example, if the K-ras StripAssay is taken to be the gold standard, then the false positives detected by this method would become false negatives detected by the other methods. To eliminate this potential ambiguity, we performed more tests to assess and compare the sensitivity thresholds of the tested methods. We used three ATCC cell lines whose *KRAS* mutation statuses are known and recorded in the COSMIC database: A549 (p.Gly12Ser), NCI-H620 (p.Gly12Val), and NCI-H2009 (p.Gly12Ala). We extracted sample DNA from the cell lines, measured its concentration by spectrophotometry, and then made dilution series of the DNA from the *KRAS* mutant cell lines in DNA from the NCI-H1975 *KRAS* wild-type cell line such that the mutant DNA comprised 25%, 20%, 15%, 10%, 5%, 1%, 0.5%, 0.25%, or 0.125% of the total *KRAS* DNA (Figure 6).

At a mutant minority of 1%, only TheraScreen and StripAssay were capable of detecting mutations in *KRAS*, while other methods have detection limit at 10% (Pyrosequencing), and 25% (HRM and Sanger sequencing). Interestingly, in one technical replicate the mutation detected by the TheraScreen DxS kit in cell line A549 (p.Gly12Cys) was inconsistent with what was actually present. At a mutant minority of 0.5%, the TheraScreen DxS kit only detected mutation in the NCI-H620 cell line (p.Gly12Val); the K-ras StripAssay failed to yield any positive results when analyzed using the StripAssay Evaluator software, but was judged to have correctly detected a mutation in the NCI-H620 line on the basis of visual inspection. At a mutant minority of 0.25%, only the K-ras StripAssay yielded a positive result. Remarkably, the K-ras StripAssay was able to detect the mutation in the NCI-H2009 line (p.Gly12Ala) even at a mutant minority of 0.125%.

## Discussion

We have examined the ability of five different methods to detect mutations in the *KRAS* gene in 131 DNA samples. *KRAS* mutations were detected in 21 samples (16.0%), 107 samples were found to contain wild-type DNA (81.7%), and three yielded inconclusive results (2.2%) (Table 1). Of the 21 samples in which mutation was detected by one or more methods, there were only four for which all five yielded a positive result (19.0%). Of the 95 wild-type samples analyzed by all five methods, concordance was observed in 87 (91.6%); overall, the five methods were in agreement with one-another for 78% of the samples examined. Excluding HRM, the four remaining analytical methods all generated positive results for 4 out of 21 samples found to be positive by one or more method (4.4%) and all generated negative results for 101 of 107 samples found to be negative by one or more method (94.4%), giving an overall agreement of 82%.

Our findings concerning the ability of these methods to detect mutations in *KRAS* are similar to those of Whitehall et al. (2009), who compared Dideoxy sequencing, HRM, the TIB Molbiol kit (Berlin, Germany), and the TheraScreen DxS (Manchester, UK) kit using DNA isolated from frozen colorectal cancer tissues. In their study, all five methods were found to be in concordance with regard to the *KRAS* mutation status of 66 of the 80 samples tested (83% agreement) [20].

Both our results and those obtained by Whitehall [20] show that a significant number of samples from colorectal tumor and NSCLC contain mixtures of *KRAS* wild-type and *KRAS* mutant cells, and that in many cases the percentage of mutant cells is below the threshold that can be detected by direct sequencing. This inherent heterogeneity of bioptic tumor tissues is an universal problem, albeit one that can be partially addressed by concentrating the tumor cells (e.g. by laser capture microdissection) before extracting their DNA. However, the fact that even a pure sample of tumor cells may contain large quantities of wild-type *KRAS* further complicates the selective identification of mutations in this gene. Consequently, it is desirable that methods for detecting *KRAS* mutations should be highly sensitive, and this point should be borne in mind when selecting a proper diagnostic method. Our study identified the TheraScreen DxS kit as having the best ability to detect *KRAS* mutations in clinical samples, followed by the K-ras StripAssay (Table 4).

**Table 4:** **Pairwise concordance between methods for *KRAS *mutation detection**

		**Direct sequencing**		**TheraScreen DxS**		**K-ras StripAssay**		**Pyrosequencing**		**HRM**	
		**+**	**-**	**+**	**-**	**+**	**-**	**+**	**-**	**+**	**-**
**Direct sequencing**	**+**			*0.338*		*0.257*		*0.735*		*0.537*	
	**-**										
**TheraScreen DxS**	**+**	**5**	**15**			*0.790*		*0.555*		*0.739*	
	**-**	**1**	**110**								
**K-ras StripAssay**	**+**	**5**	**21**	**19**	**7**			*0.438*		*0.500*	
	**-**	**1**	**104**	**1**	**104**						
**Pyrosequencing**	**+**	**6**	**4**	**9**	**1**	**9**	**1**			*0.687*	
	**-**	**0**	**121**	**11**	**110**	**17**	**104**				
**HRM**	**+**	**6**	**9**	**12**	**3**	**11**	**4**	**9**	**6**		
	**-**	**0**	**99**	**4**	**95**	**12**	**87**	**1**	**98**		

Every intersection of method row and method column corresponds to a 2x2 contingency table for two methods. The upper right part of the table is filled with κ concordance metrics.

Our results also indicate that direct sequencing is only of limited utility when trying to detect mutations in the *KRAS* gene in cancer tissues, since this method only detected *KRAS* mutations in 6 of the 131 DNA samples tested, even though 21 were found to contain mutations by other methods. Though direct sequencing is still being advocated as *KRAS* genotyping method of choice [21], it missed 72% of all mutations in our cohort. Obviously, the sensitivity of the sequencing methods could be further improved by using laser microdissection [22], preferential preamplification [23], inclusion of both primary and metastatic tissues in the analysis, or by using clamping to suppress PCR amplification of the wild-type gene [24]. However, we agree with Pinto *et al*. that Sanger sequencing (without the first steps of COLD-PCR) [25] is currently outperformed by more sensitive techniques [26].

Pyrosequencing is easily capable of detecting PCR fragments that are 25–50 bp in length while longer fragments may pose a problem. However, this is not the case of detecting mutations in *KRAS*, because the most frequent mutations in this gene are adjacent, occurring in codons 12 and 13. It may even be advantageous to use short fragments when diagnosing mutations because DNA may be fragmented during the processing of clinical tissue samples. In accordance with results of others [27,28], Pyrosequencing outperformed conventional sequencing for detecting *KRAS* mutations in samples with levels of mutant cells ranging from 5 to 25% (Table 4) while quantification of mutated portion of DNA was not possible. This is probably due to preferential amplification of the mutated samples by the primers designed for the particular Biotage kit used. This shortcoming could be obviated by a better primer design or other modification of the kit and/or improvements in the interpretation algorithm [29,30]. Promisingly, a massively parallel pyrosequencing system using nanoliter reaction volumes has yielded satisfying results in an interlaboratory comparison [28]. While this probably represents the future of testing in predictive oncology, such systems are prohibitively costly for most laboratories at the present.

HRM proved to be the least expensive and the most rapid method, as it requires only standard real-time PCR reagents and a slightly prolonged PCR protocol. Despite the optimistic references from other laboratories [31], the analysis of the melting profiles in our hands remains less reliable than other methods, and even repeated testing of our reference DNA did not always yield consistent results. Because of this, the typing of two samples by this method was inconclusive. We may speculate with Do [32] that treatment of DNA with uracil glycosylase or special step of DNA cleaning would help standardize the method and better its analytical parameters. Interestingly, HRM analysis identified mutations in the *KRAS* locus of two DNA samples (samples 31 and 32) for which none of the other methods detected any mutation (Table 1). In keeping with the findings of other authors [33], we interpret these results as reflecting a tendency of HRM to generate false positives. However, it is possible that they reflect rare mutations outside codon 12 and 13 that destabilize heteroduplex DNA even in the presence of an excess of wild-type DNA. Although cost and time efficiency are important factors in clinical diagnosis, the reproducibility of the HRM method will need to be improved before it can be considered viable. This could potentially be done by changing primers [34], adding melting standards [35], spiking with oligonucleotides [36], or combination with SNaPshot [37].

The StripAssay was the most analytically sensitive test (Table 2) of those we examined. On the basis of the results obtained with this method in the series of tests conducted with dilution series of mutant *KRAS* DNA (Figure 6), one could even argue that samples 24 to 30 should be reassigned as mutants (Table 2), thereby changing the false positive rate for the K-ras StripAssay to 0/128 and the false negative rate for TheraScreen DxS to 7/128. However, the interpretation of StripAssay results can be quite problematic for samples whose mutant DNA content is below 1% (see the result obtained with a mutant minority of 0.5% NCI-H620 in Figure 6). Insofar, it was not tested in clinical studies what is a significance of fraction of mutated cells below 1%, regardless of the typing method used.

During time of submitting this article, company’s software was upgraded to follow more precisely the requirements of ISO15189 norm (scanner calibration standard was added and manual baseline correction feature was removed). It remains to be seen if such changes bring any improvement to diagnostic accuracy.

Of the methods examined in this study, the TheraScreen DxS kit was the fastest method and exhibited the highest sensitivity and specificity. However, it was also the most expensive method that is not free of false reactions. Specifically, the kit failed to detect the p.Gly13Cys mutation in sample 2 because it is not designed to detect this mutation. Although the frequency of the mutations that are not covered by the TheraScreen DxS test is very low and clinically not highly relevant, this nevertheless constitutes an inherent limitation of the kit. In addition, the precise allelic mutation detected by this kit in samples 3, 16, and 18 differed from the consensus result. While this could potentially be due to stochastic variation in the early events of PCR priming, there is no firm evidence to support this hypothesis. Although discrepancies in the precise identity of the mutation are not yet clinically relevant, and these results were not scored as errors in this study, this finding warrants caution when using the ARMS Scorpions assay in different diagnostic setups, where the type of mutation is important (e.g. when looking at the T790M and S768I activating mutations in *EGFR* genotyping). As discussed above, samples 24 to 30 gave positive results in the StripAssay but were negative when analyzed with the TheraScreen DxS kit, and they seem to have a mutant population in the clinically “grey area,” having less than 1% of the cells in the sample containing *KRAS* mutation. Ideally, their status should have been resolved by PCR amplicon cloning, followed by sequencing of the clones, digital PCR, or ultradeep sequencing. However, this approach is not practical for routine work and we did not have sufficient DNA to perform this experiment. Moreover, the low frequency of *KRAS* mutations in patient tumors have unknown clinical relevance, since all drug registration trials were performed using 1% of mutant *KRAS* cells as a low detection limit of the method.

The properties of the different methods examined in this work are summarized in Table 5.

**Table 5:** Summary of the properties of the different methods

	**Sanger sequencing**	**Pyrosequencing**	**TheraScreen DxS**	**StripAssay**	**HRM**	
**CE mark**	no	no	yes	yes	no	**CE mark**
Limit of detection*	25-30 %*	5-10 %*	1 %	below 1 %	5-10 %*	Limit of detection*
**Turnaround time**	2-3 days	2 days	1/2 day	1 day	1/2 day	**Turnaround time**
**Ease of interpretation**	easy	easy	easy	medium	difficult	**Ease of interpretation**
**Technician time**	6 hrs	4 hrs	2 hrs	5 hrs	2 hrs	**Technician time**
**Amount of input DNA**	1 reaction	1 reaction	8 reactions	1 reaction	1 reaction	**Amount of input DNA**
**Detection of rare mutations**	Yes – can detect any mutation located between the primers.	Yes – can detect any mutation within the short sequencing fragments.	No – can only detect 7 specific mutations.	No – can only detect 10 specific mutations.	Yes – can detect some mutations located between the primers.	**Detection of rare mutations**
**Reagent cost**	20 €	40 €	120 €	60 €	4 €	**Reagent cost**
**Special equipment required**	Sequencer 70 000 €	Pyrosequencer 150 000 €	Real time PCR cycler 30 000 €	PCR cycler 7 500 €	HRM Real time PCR cycler 75 000 €	**Special equipment required**

* from reference of Tsiatis^26^ and Ogino^27^.

We agree with Tsiatis et al. [27] that for research purposes more than one genotyping platform is necessary to reveal double mutations and to provide complementary data. In clinical settings, the most readily accessible NSCLC sample type is needle or brush biopsy, which is examined cytologically while resected, or biopsied tumors processed by formaldehyde fixation and paraffin embedding (FFPE). Proportion of FFPE samples from all samples usually reflects the best local practice and experience. Unfortunately, the FFPE process alters significantly the quality of DNA, and in many cases the DNA isolation from cytology smears yields higher quality albeit lower quantity of DNA.Very low quantity of available DNA isolated from cytological preparations was a major limiting factor in our comparative study, which we tried to overcome using frozen tissue from biobank, since it provides both high quality and quantity of DNA. Moreover, due to recent biobanking initiatives [38], we are more frequently facing situations, where the tumor molecular diagnostics is performed from frozen tissues. Of the 11 FFPE samples genotyped using both the StripAssay and TheraScreen, 5 samples could not be typed by at least one method, 2 samples were wildtype by both methods, 3 samples were mutant by both methods, and one sample was p.Gly12Asp by TheraScreen and wildtype by StripAssay. From one point of view, it could be argued that our genotyping results obtained using frozen samples are transferable to genotyping of FFPE samples because the mechanisms by which the methods work are not dependent on the nature of the input sample. On the other hand, it should be noted that improperly-performed paraffin embedding damages DNA and can favor methods that are more robust to variation in the amount and quality of the starting material (this would arguably disfavor TheraScreen because it requires eight PCR reactions whereas the other methods require only one equivalent reaction). It has been suggested that the issue of limited material for testing can be largely circumvented by using whole genome amplification techniques [39,40], although the potentially biasing impact of the genome amplification techniques on low frequency somatic mutation genotyping is still not fully addressed. However, we suppose that our tests of kit performance on frozen tissue samples provide useful insights into their general utility and will be valuable for orchestrating genotyping efforts across molecular pathology laboratories.

## Conclusions

The performance of five methods (Direct sequencing, Pyrosequencing, High resolution melting analysis, the TheraScreen DxS kit, and the K-ras StripAssay) for detecting mutations in the *KRAS* gene was compared using DNA extracted from 131 frozen NSCLC samples. The TheraScreen DxS kit was found to be the most effective, followed by the StripAssay kit. However, because of the heterogeneity of typical cancer tissue samples and the differences in the two methods’ mechanisms of action, there are still unsatisfactory numbers of discrepancies between these two ‘best’ methods, which failed to agree on 8 of the 131 specimens examined in this work. Nevertheless, our findings should facilitate the rational selection of methods for detecting mutations at the *KRAS* locus using heterogeneous clinical samples obtained from biopsies of cancer patients.

## Abbreviations

ASCO: American Society of Clinical Oncology; ATCC: American Tissue Culture Collection; CE: Conformité Européenne; ESP: European Society of Pathology; FFPE: Formalin-fixed paraffin embedded; HRM: High resolution melting; KRAS: Ki-ras2 kristen rat sarcoma viral oncogene homolog; mCRC: Metastatic colorectal cancers; NCCN: U.S. National Comprehensive Cancer Network; NSCLC: Non-small cell lung cancer; SNP: Single nucleotide polymorphism.

## Competing interests

The author(s) declare that they have no competing interests.

## Author’s contributions

SJ: carried out the preparation of the samples and molecular genetic testing (pyrosequencing, TheraScreen assay, StripAssay, and HRM) and drafted the manuscript, JD: validated TheraScreen and StripAssay, interpreted HRM assays, revised the manuscript critically for important intellectual content, JB: carried out the molecular genetic testing (sequencing) and drafted the manuscript, YX: contributed in preparation of samples and carried out the molecular genetic testing analysis (pyrosequencing) and drafted the manuscript, MS: carried out the molecular genetic testing (TheraScreen assay) and drafted the manuscript, JK: surgically sampled patients and drafted the manuscript, VK: took care for patients and provided clinical data and drafted the manuscript, JŠ: carried out immunohistopathological testing to confirm disease status and drafted the manuscript, TT: carried out immunohistopathological testing to confirm disease status and drafted the manuscript, IG: took care for patients, provided and analysed clinical data, DR: concepted and designed the study, interpreted and analysed the data, MH: concepted and designed the study, interpreted and analysed the data, revised the manuscript critically for important intellectual content. All authors read and approved the final manuscript.
